# Peculiarities of the microwave properties of hard–soft functional composites SrTb_0.01_Tm_0.01_Fe_11.98_O_19_–AFe_2_O_4_ (A = Co, Ni, Zn, Cu, or Mn)

**DOI:** 10.1039/d0ra05087c

**Published:** 2020-09-03

**Authors:** A. V. Trukhanov, N. A. Algarou, Y. Slimani, M. A. Almessiere, A. Baykal, D. I. Tishkevich, D. A. Vinnik, M. G. Vakhitov, D. S. Klygach, M. V. Silibin, T. I. Zubar, S. V. Trukhanov

**Affiliations:** SSPA “Scientific and Practical Materials Research Centre of NAS of Belarus” Minsk 220072 Belarus truhanov86@mail.ru; South Ural State University Chelyabinsk 454080 Russia; Department of Biophysics, Institute for Research and Medical Consultations (IRMC), Imam Abdulrahman Bin Faisal University P.O. Box 1982 31441 Dammam Saudi Arabia; Department of Physics, College of Science, Imam Abdulrahman Bin Faisal University P.O. Box 1982 Dammam 31441 Saudi Arabia; Department of Nanomedicine Research, Institute for Research and Medical Consultations (IRMC), Imam Abdulrahman Bin Faisal University P.O. Box 1982 31441 Dammam Saudi Arabia; Ural Federal University Ekaterinburg 620002 Russia; Institute of Advanced Materials and Technologies, National Research University of Electronic Technology “MIET” 124498 Zelenograd Moscow Russia; Institute for Bionic Technologies and Engineering, I.M. Sechenov First Moscow State Medical University Moscow 119991 Russia; Scientific-Manufacturing Complex “Technological Centre” 124498 Zelenograd Moscow Russia

## Abstract

Herein, we investigated the correlation between the chemical composition, microstructure, and microwave properties of composites based on lightly Tb/Tm-doped Sr-hexaferrites (SrTb_0.01_Tm_0.01_Fe_11.98_O_19_) and spinel ferrites (AFe_2_O_4_, A = Co, Ni, Zn, Cu, or Mn), which were fabricated by a one-pot citrate sol–gel method. Powder XRD patterns of products confirmed the presence of pure hexaferrite and spinel phases. Microstructural analysis was performed based on SEM images. The average grain size for each phase in the prepared composites was calculated. Comprehensive investigations of dielectric properties (real (*ε*′) and imaginary parts (*ε*′′) of permittivity, dielectric loss tangent (tan(*δ*)), and AC conductivity) were performed in the 1–3 × 10^6^ Hz frequency range at 20–120 °C. Frequency dependency of microwave properties were investigated using the coaxial method in frequency range of 2–18 GHz. The non-linear behavior of the main microwave properties with a change in composition may be due to the influence of the soft magnetic phase. It was found that Mn- and Ni-spinel ferrites achieved the strongest electromagnetic absorption. This may be due to differences in the structures of the electron shell and the radii of the A-site ions in the spinel phase. It was discovered that the ionic polarization transformed into the dipole polarization.

## Abstract 2

Paper presents the correlation between the composition, microstructure, and microwave properties of composites based on Tb/Tm-doped Sr-hexaferrites and spinel ferrites (AFe_2_O_4_), which were fabricated by a one-pot citrate sol–gel method.

## Graphical abstract

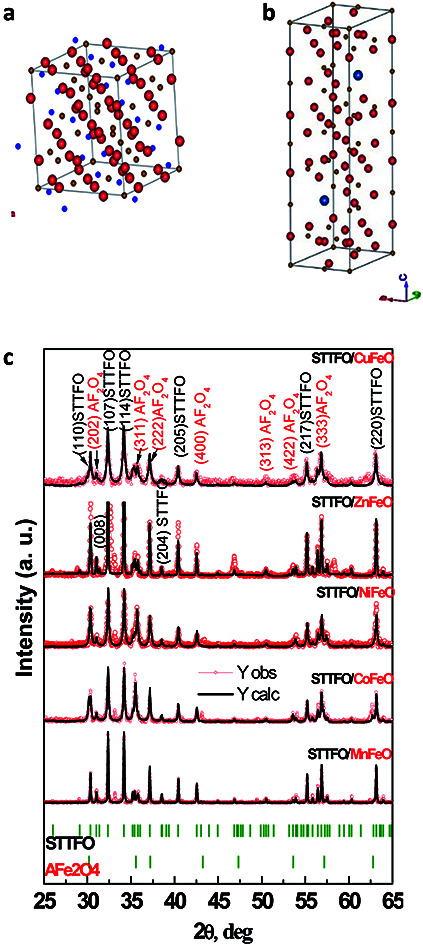

## Introduction

Highly interdependent transition metal oxides demonstrate a broad range of uncommon phenomena, which can be used for practical applications.^1–3^ Electrical and magnetic characteristics are result of the collaborative effects of charge and spin ordering. These materials show different quantum effects, for example, Bose–Einstein condensation of magnons, high-temperature superconductivity, and multiferroicity (the coexistence of magnetic and ferroelectric ordering). Currently, functional materials with coexisting soft and hard magnetic properties at room temperature have attracted significant interest, among which multiferroic and electromagnetic composites are most noteworthy. A strong coupling between them may be caused by the coexistence of two separate magnetic phases, resulting in more advanced functional properties; for instance, it may cause an alteration of the original electrical and magnetic characteristics compared to those of pure materials. This study was aimed to identify a relationship between the chemical composition (concentration ratio of different phases) and functional properties of composites. Most interesting effects can be observed in magnetic composites with soft and hard magnetic phases due to internal strong coupling exchange.

Many researchers have concentrated on complex metal oxides that are based on Fe ions. The most interesting materials for researchers are barium M-type hexaferrite (AFe_12_O_19_, where A = Ba, Sr, Pb) and solid solutions based on these complex metal oxides. These compounds possess a magnetoplumbite structure and the space group *P*6_3_/*mmc* (no. 194) with cell parameters *a* = *b* ≈ 5.90 Å and *c* ≈ 23.30 Å. Owing to their high saturation magnetization, low electrical conductivity, and large magnetocrystalline anisotropy, M-type hexaferrites are of great importance for microwave applications.^4^ Microwave absorption in AFe_12_O_19_ occurs by two main processes: (1) domain boundary resonance and (2) natural ferromagnetic resonance. Microwave absorbers attract much attention due to broad prospects of the practical applications (weakening of the transmitted electromagnetic radiation).^5–8^ Hard and soft nanocomposites are technologically important materials because of their specific applications in magnetic recording media, magnetic fluids, microwave devices, permanent magnets, and biomedicines.^9,10^ These magnetic composites achieve high coercivity from the M-type hexaferrite materials (hard ferrite) and high saturation magnetization from the spinel ferrites (soft ferrite), and exhibit outstanding magnetic performance called exchange-spring magnets. The exchange-coupled systems depend on several parameters such as phase distribution, phase composition, crystallite size of each phase, and magnetic interactions. Since there is a strong exchange-coupling between the hard and soft magnetic phases, the microwave absorption of the composite is amplified.^11–16^ Moreover, several studies have been conducted to improve the magnetic characteristics, specifically the exchange-coupling behavior by joining substitution hexaferrite with spinel ferrite. Mansour *et al.* have explored the dielectric and magnetic properties of *x*(BaFe_11.7_Al_0.15_Zn_0.15_O_19_)/1 − *x*(Mn_0.8_Mg_0.2_Fe_2_O_4_), where *x* = 0.3, 0.4, or 0.5, prepared by a sol–gel combustion approach and noticed an improvement in the magnetic properties of this composite.^17^ Almessiere *et al.* have illustrated the magnetic characteristics of Sr_0.3_Ba_0.4_Pb_0.3_Fe_12_O_19_/(CuFe_2_O_4_)_*x*_ (hard/soft) composite, where *x* = 1, 2, 3, 4, or 5.^18^ They found an enhancement in the magnetization of the composite and a strong exchange-coupling behavior. Recently, we examined exchange-coupling effects in a series of SrTb_0.01_Tm_0.01_Fe_11.98_O_19_/AFe_2_O_4_ hard/soft nanocomposites, in which A can be Zn, Co, Cu, Ni, or Mn.^19^ Their morphological, structural, and magnetic properties were comprehensively investigated. The differently prepared composites presented an achievement exchange-coupling effect in one-step. Among the various prepared composites, the SrTb_0.01_Tm_0.01_Fe_11.98_O_19_/CoFe_2_O_4_ nanocomposite exhibited the best exchange-coupling behavior along with the highest *M*_r_, *M*_s_, and *H*_c_ values. The benefits of exchange coupling ferrites were discovered by Shen *et al.*^20^ Herein, a study on the SrTb_0.01_Tm_0.01_Fe_11.98_O_19_/AFe_2_O_4_ (A = Co, Ni, Zn, Cu, or Mn) functional composites based on SrTb_0.01_Tm_0.01_Fe_11.98_O_19_ hexaferrites as the hard magnetic phase and AFe_2_O_4_ ferrite spinel as the soft magnetic phase with different compositions (different A-site ions) was performed *via* microstructural and microwave analysis. In this study, the interrelation between the structure and the microwave properties of these composites was investigated.

## Experimental

### Synthesis

The SrTb_0.01_Tm_0.01_Fe_11.98_O_19_/AFe_2_O_4_ (A = Co, Ni, Zn, Cu, or Mn) functional composites based on SrTb_0.01_Tm_0.01_Fe_11.98_O_19_ hexaferrites as the hexagonal phase and AFe_2_O_4_ ferrite spinel as the cubic magnetic phase with different compositions (different A-site ions) were produced using a one-pot sol–gel auto-combustion method.^21–24^ The solutions of the hard and soft magnetic phases were separately synthesized. For the hard magnetic phase SrTb_0.01_Tm_0.01_Fe_11.98_O_19_ (STTFO), the initial reagents Sr(NO_3_)_2_ (strontium nitrate), Fe(NO_3_)_3_·9H_2_O (iron nitrate), Tm_2_O_3_ (thulium oxide), and Tb_4_O_7_ (terbium oxide) were mixed in a stoichiometric molar ratio (1 : 0.01 : 0.01 : 11.98) followed by the addition of citric acid and deionized water at 355 K. For the soft magnetic phase AFe_2_O_4_ or AFO (A = CoFO, NiFO, ZnFO, CuFO, or MnFO), the nitrates of nickel (Ni(NO_3_)_2_·6H_2_O), cobalt (Co(NO_3_)_2_·6H_2_O), copper (Cu(NO_3_)_2_·6H_2_O), zinc (Zn(NO_3_)_2_·6H_2_O), and manganese (Mn(NO_3_)_2_·6H_2_O) were mixed in a stoichiometric molar ratio (1 : 2). Finally, the resulting compositions of the hard and soft phases were mixed together with the slow addition of citric acid under stirring at 90 °C. The pH of the mixtures was regulated at 7 by adding an NH_3_ solution; after this, the mixture was heated at 150 °C for 30 min, and then, the temperature was increased to 330 °C for the formation of a viscous gel. Ignition of the gel occurs when a huge amount of gas is formed during combustion along with the formation of a black-coloured powder. To obtain the required hard/soft composites, the resulting powders were sintered at a temperature of 1000 °C for 5 hours. The following five functional ferrite-based composites were obtained and systematically investigated:

(1) SrTb_0.01_Tm_0.01_Fe_11.98_O_19_/CoFe_2_O_4_ (STTFO/CoFO);

(2) SrTb_0.01_Tm_0.01_Fe_11.98_O_19_/NiFe_2_O_4_ (STTFO/NiFO);

(3) SrTb_0.01_Tm_0.01_Fe_11.98_O_19_/ZnFe_2_O_4_ (STTFO/ZnFO);

(4) SrTb_0.01_Tm_0.01_Fe_11.98_O_19_/CuFe_2_O_4_ (STTFO/CuFO); and

(5) SrTb_0.01_Tm_0.01_Fe_11.98_O_19_/MnFe_2_O_4_ (STTFO/MnFO).

### Structural investigations

The specificities of the crystal structure and phase compositions were studied by XRD using Cu-Kα radiation (Rigaku D/MAX-2400, Japan). The chemical compositions and microstructures were investigated by SEM (Hitachi S-4800, Japan) with EDX. The particle size distribution of the composites was examined by the analysis of the images obtained using SEM. The proportion of the particle area *P*_i_ was determined for each fraction using the following equation:1
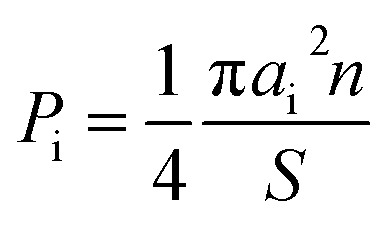
where *a*_i_ is the size of a certain particle (or diameter of a disk that has size equal to the size of particles), *S* is the total analyzed area, and *n* is number of particles with a specified size.

The specific surface area (SSA) of one gram of composite [m^2^ g^−1^] was calculated as follows:2
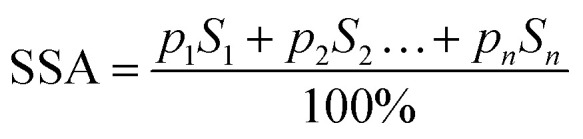
where *p*_i_ is the content of each fraction [%] and *S*_i_ is the surface area of the particles of a certain fraction, which was obtained as3
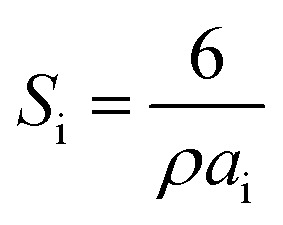
where *ρ* is the radiographic or XRD density of composites [g cm^−3^] that was calculated using the following equation:4
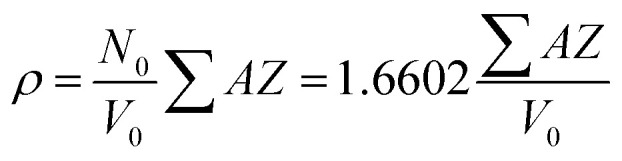
where *N*_0_ is the Avogadro constant (6.02214 × 10^23^ mol^−1^), *A* is the atomic weight of each component in the composite, and *Z* is the number of atoms in a unit cell.

### Microwave investigation

The permeability and permittivity of the composites with respect to frequency were studied by the coaxial method using an Agilent network analyzer in the frequency range of 2–18 GHz. The coaxial line impedance was normalized (*Z* = 50 ohm). Initially, total two-port calibration was carried out on the test setup. This is necessary to eliminate errors related to source and load matching, directivity, isolation, and frequency response in both the forward and reverse measurements. The inner and outer conductor diameters of the measuring cell used for the coaxial method were 3 mm and 7 mm, respectively. The height of the investigated sample was 10 mm. For testing, the samples were prepared in the form of a hollow cylinder for a snug fit to the measuring cell. The obtained values of the reflection (*S*_11_) and transmission (*S*_21_) coefficients of the samples were converted into the real and imaginary parts of the permeability (*μ̇*′ and *μ̇*′′) and permittivity (*ε̇*′ and *ε̇*′′), respectively. The permeability of the material was then established using the Nicolson–Ross–Weir algorithm from the *S*-parameters obtained as a function of frequency.^25,26^ More information about the experimental setup can be found in the literature.^27^

## Results and discussions

### Crystal structure and microstructure

**Fig. 1:**
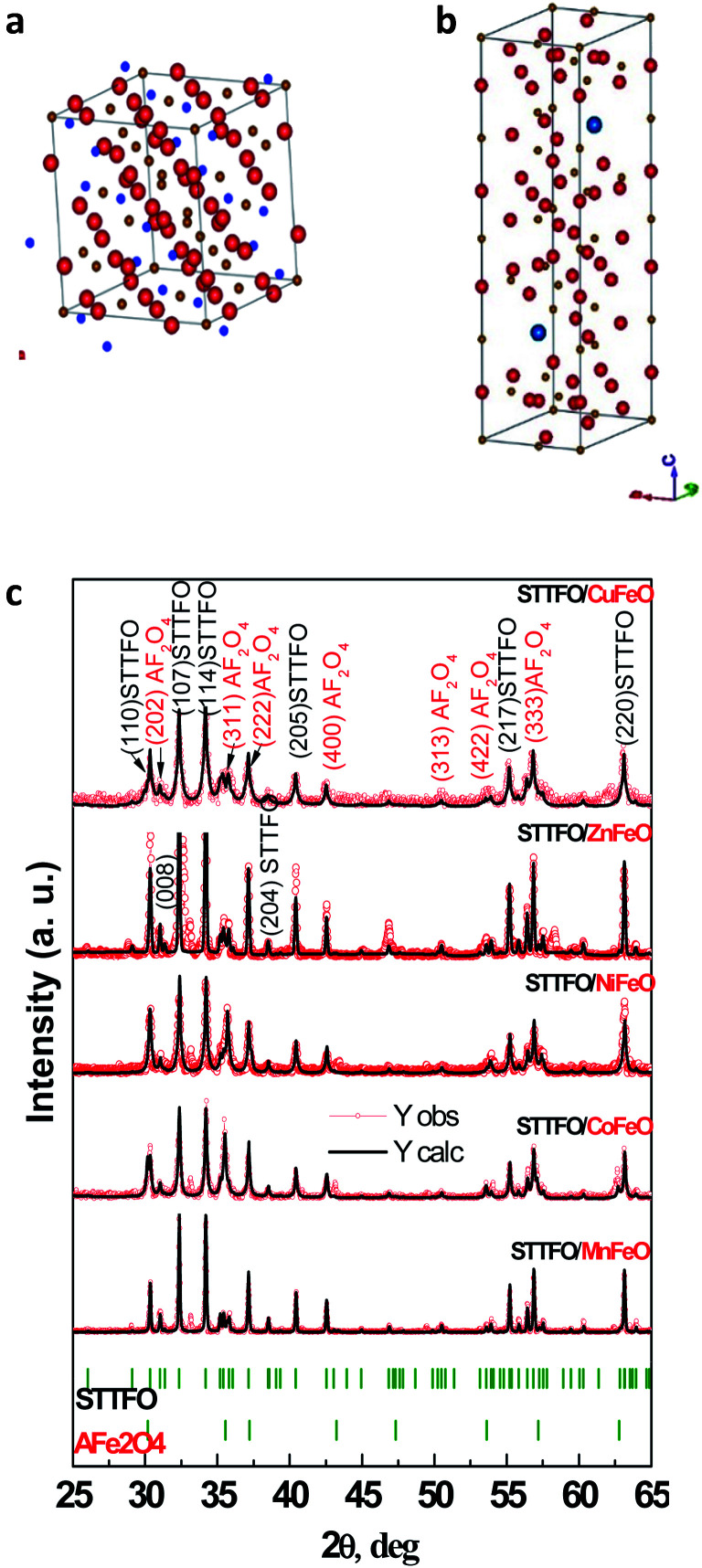
Models of the unit cells of the (a) spinel structure, SG: *Fd*3̄*m* and (b) M-type hexaferrite structure, SG: *P*6_3_/*mmc* and the XRD patterns (c) of all the STTFO/AFe_2_O_4_ samples.

All samples have been previously investigated by XRD^28^ (Fig. 1c). A peak corresponding to an unimportant impurity phase resembling Fe_2_O_3_ was observed. According to Table 1, all the samples mainly contain two major phases: a soft phase (AFe_2_O_4_) with a cubic structure (corresponding to spinels with the space group *Fd*3̄*m*, as shown in Fig. 1a) and a hard phase (AFe_12_O_19_) with a magnetoplumbite structure (corresponding to M-type hexaferrites with the space group *P*6_3_/*mmc*, as shown in Fig. 1b).

**Table 1:** Unit cell parameters of the STTFO/AFe_2_O_4_ (A = Co, Ni, Zn, Cu, or Mn) functional composites

Composition	*χ* ^2^	AFe_2_O_4_		SrTb_0.01_Tm_0.01_Fe_11.98_O_19_		
		*a* (Å)	*V* (Å^3^)	*a* (Å)	*c* (Å)	*V* (Å^3^)
STTFO/CoFO	1.71	8.3897	590.526	5.8822	23.0299	690.064
STTFO/NiFO	1.49	8.3410	580.302	5.8839	23.0347	690.607
STTFO/ZnFO	1.97	8.3319	578.405	5.8854	23.0420	691.178
STTFO/CuFO	2.15	8.3414	580.386	5.8883	23.0502	692.106
STTFO/MnFO	2.03	8.3270	577.385	5.8843	23.0268	690.464

After this, the radiographic density and SSA values of the composite samples were determined. The results are provided in Table 3.

It was found that the STTFO/CoFO composite has the largest particle size (1.01 μm) and the smallest SSA value (about 1 141 000 m^2^ g^−1^). The maximum value of SSA corresponds to the STTFO/CuFO composite and is equal to 1 403 000 m^2^ g^−1^.

**Fig. 2:**
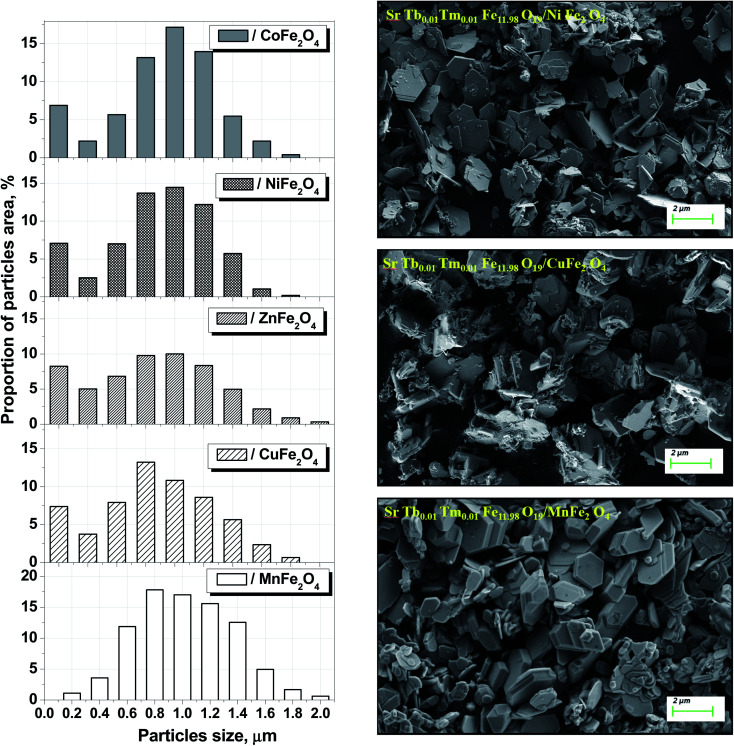
Size distribution and SEM images of the composite particles for each STTFO/AFe_2_O_4_ (A = Co, Ni, Zn, Cu, or Mn) functional composite.

We have successfully described the structures of all the composite samples as hard (similar to the magnetoplumbite structure, SG: *P*6_3_/*mmc*, no. 194) and soft (cubic, SG: *Fd*3̄*m*, no. 227) phases. The low values of the fitting parameter – *χ*^2^ (goodness-of-fit quality factor) suggest that the studied samples are of better quality and the refinements of neutron data are effective. Rietveld refinement using the FullProf software was used to calculate the main structural parameters: lattice constant (*a*), volume of the unit cell (*V*), and average crystallite sizes. The main structural parameters (lattice constants and average crystallite size) of the composites are presented in Table 1. The data correlates well with the differences in the ionic radii of the A-sites in spinels. Using the SEM images (Fig. 2) of the products, we calculated the average grain (or particle) sizes (Fig. 2).

A particle consists of nanosized crystallites. The average crystallite size (*D*_XRD_) was determined using the XRD data, and the average particle size was determined *via* the SEM images. The particle size distributions for the composites are shown in Fig. 2.

The distribution of the STTFO/MnFO sample corresponds to the Gaussian-type function. In other words, there is only one most probable value of the particle size (or average particle size). This value was found to be the maximum of the Gaussian function and is equal to 954 nm for the STTFO/MnFO sample. The SEM image of the STTFO/MnFO composite confirms the uniform Gaussian distribution.

**Fig. 3:**
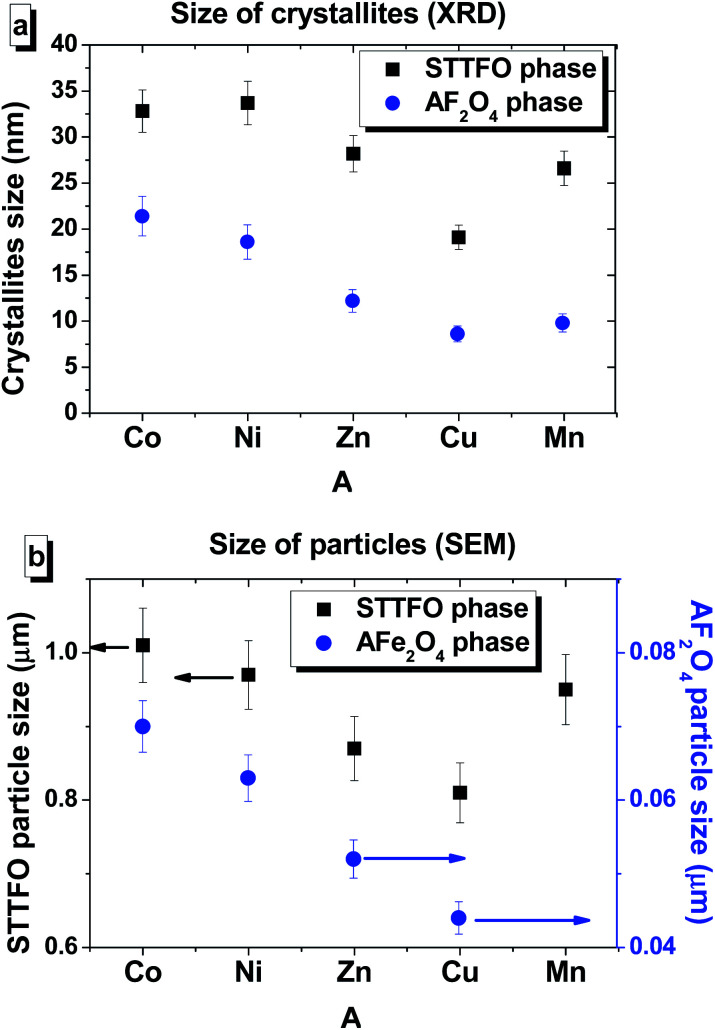
The values of the average crystallite size (obtained using the XRD data) (a) and most probable particle size (obtained using the SEM images) for the (b) STTFO (black square) and AFe_2_O_4_ (blue circle) phases of each STTFO/AFe_2_O_4_ (A = Co, Ni, Zn, Cu, or Mn) functional composite.

Other composites, except for the STTFO/MnFO composite, have a bimodal distribution. There are two extreme points of particle size in the graphs. The first extreme point (about 0.1–0.2 μm) corresponds to the contribution of the soft phase. The other extreme point (about 0.8–1.2 μm) corresponds to the contribution of the hard phase or SrTb_0.01_Tm_0.01_Fe_11.98_O_19_. Therefore, the two most probable particle sizes should be obtained for the composites containing CoFe_2_O_4_, NiFe_2_O_4_, ZnFe_2_O_4_ or CuFe_2_O_4_ components. More information about the most probable particle sizes for the hard and soft phases is presented in Fig. 3. The results of the particle size investigations conducted using the SEM images correlate well with the crystallite sizes obtained using the XRD data.

The calculation of the SSA values was carried out using eqn (2) and was based on the calculations of the theoretical or also called radiographic density according to eqn (4). The intermediate calculation results, such as the weight of the unit cell and radiographic density of the components, for the separated components of the composites are provided in Table 2.

**Table 2:** The calculated values of the weight of the unit cell and radiographic density of each component of the STTFO/AFe_2_O_4_ (A = Co, Ni, Zn, Cu, or Mn) functional composites

Composite component	Weight unit cell	Density (g cm^−3^)
SrTb_0.01_Tm_0.01_Fe_11.98_O_19_	2127.83	5.12
CoFe_2_O_4_	1876.96	5.28
NiFe_2_O_4_	1875.20	5.36
ZnFe_2_O_4_	1928.48	5.53
CuFe_2_O_4_	1913.84	5.47
MnFe_2_O_4_	1884.88	5.41

**Table 3:** The calculated values of the radiographic density and specific surface area of the STTFO/AFe_2_O_4_ (A = Co, Ni, Zn, Cu, or Mn) functional composites

Composition	Density (g cm^−3^)	SSA (m^2^ g^−1^)
STTFO/CoFO	5.34	1 141 000
STTFO/NiFO	5.36	1 177 000
STTFO/ZnFO	5.40	1 298 000
STTFO/CuFO	5.38	1 403 000
STTFO/MnFO	5.35	1 194 000

### Dielectric properties

**Fig. 4:**
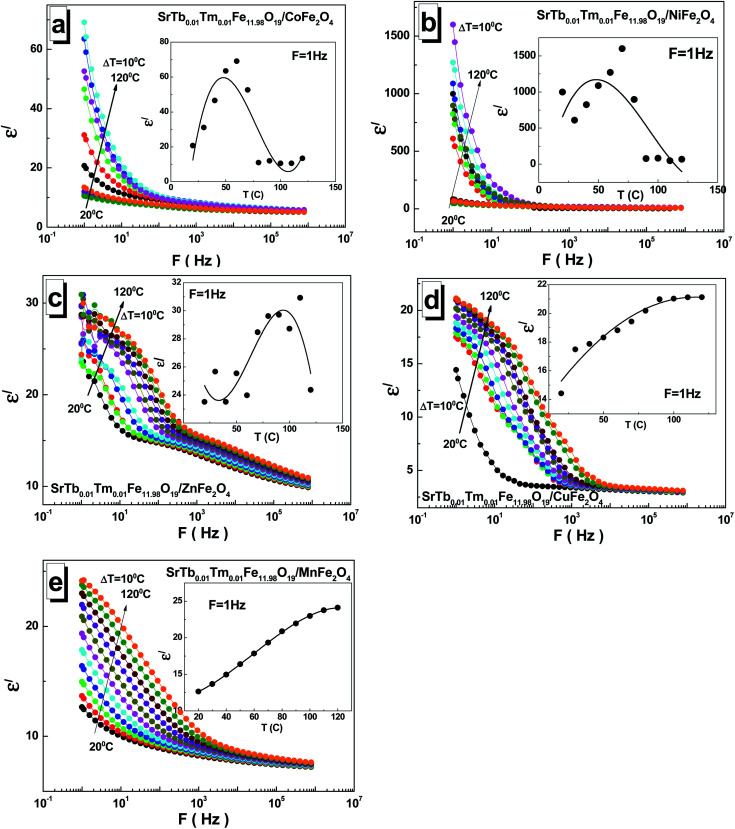
Frequency dependences of the real part *ε*′ of permittivity in the range of 1–0.8 × 10^6^ Hz at different temperatures ranging from 20 °C to 120 °C with a step of Δ*T* = 10 °C for the STTFO/AFe_2_O_4_ functional composites, where A = Co (a), Ni (b), Zn (c), Cu (d), and Mn (e). Insets demonstrate the temperature dependence of *ε*′ at *F* = 1 Hz.

To investigate the dielectric properties of the obtained STTFO/AF_2_O_4_ composite samples, the real part *ε*′ and imaginary part *ε*′′ of the complex permittivity *ε* = *ε*′ + i*ε*′′ and conductivity *C* at the alternating current in the 1–8 × 10^5^ Hz frequency and 20–120 °C temperature ranges were measured. As is generally known, the dielectric properties of ferrites are defined by their own stoichiometry, impurities, crystal structure, ceramic morphology, external signal frequency, temperature, and even humidity.^29^ To avoid the influence of moisture, the sample at the beginning of the measurement cycle was preheated to 120 °C and maintained at this temperature for 10 min; after this, it was cooled to the required measurement temperature. The frequency dependence of the real part *ε*′ of permittivity at different temperatures for all the STTFO/AF_2_O_4_ composites is shown in Fig. 4(a–e).

A relaxation process was clearly observed for all the samples at all measured temperatures, demonstrating normal ferrimagnetic behavior.^30^ At low frequencies, a high dispersion of the real part *ε*′ of the dielectric constant was observed. It remained practically unchanged at high frequencies, as has been reported previously.^31^ The low-frequency value monotonically depends on temperature only for the samples based on Mn^2+/3+^ and Cu^2+^ cations (see the insets of Fig. 4(a–e)). In these cases, the low-frequency value increases. For the samples based on Ni^2+/3+^ cations, a maximum was obtained in the region of 50–80 °C. For the STTFO/AF_2_O_4_ samples based on Co^2+/3+^ and Zn^2+^ cations, an inflection point was noticed in the region of 50–80 °C. The maximum value of ∼1600 for the real part *ε*′ of permittivity was obtained at 1 Hz and 70 °C for the sample based on the Ni^2+/3+^ cations. The minimum value of ∼11 for the real part *ε*′ of permittivity was achieved at 1 Hz and 100 °C for the sample based on the Co^2+/3+^ cations.

For the STTFO/AF_2_O_4_ composite samples studied herein, the relaxation of the real part *ε*′ of permittivity in the low-frequency range can be considered to be due to the polarization of the grain boundaries. With the increasing temperature, additional polarization of the sample surface was also noticed at low frequencies. This behavior is associated with the accumulation of charge carriers at the electrode–sample interface.^32^ The polarization of the electrodes and grain boundaries at low frequencies can be defined as the Maxwell–Wagner polarization.^33^ The diffusion of the charges accumulated at the inhomogeneous grain boundaries requires significantly more energy in the low-frequency region than the tunnelling of carriers at high frequencies.^34^ A local charge displacement, due to which the polarization of the inhomogeneous boundaries occurred, was caused by the exchange of electrons between Fe^2+^ and Fe^3+^ cations in the structure of ferrite. In addition, these carrier motions at higher frequencies can contribute to the formation of small or large polarons.^35–37^ Using the analysis of a complex electric module, the role of the grains and grain boundaries in these processes can also be characterized.^38^

**Fig. 5:**
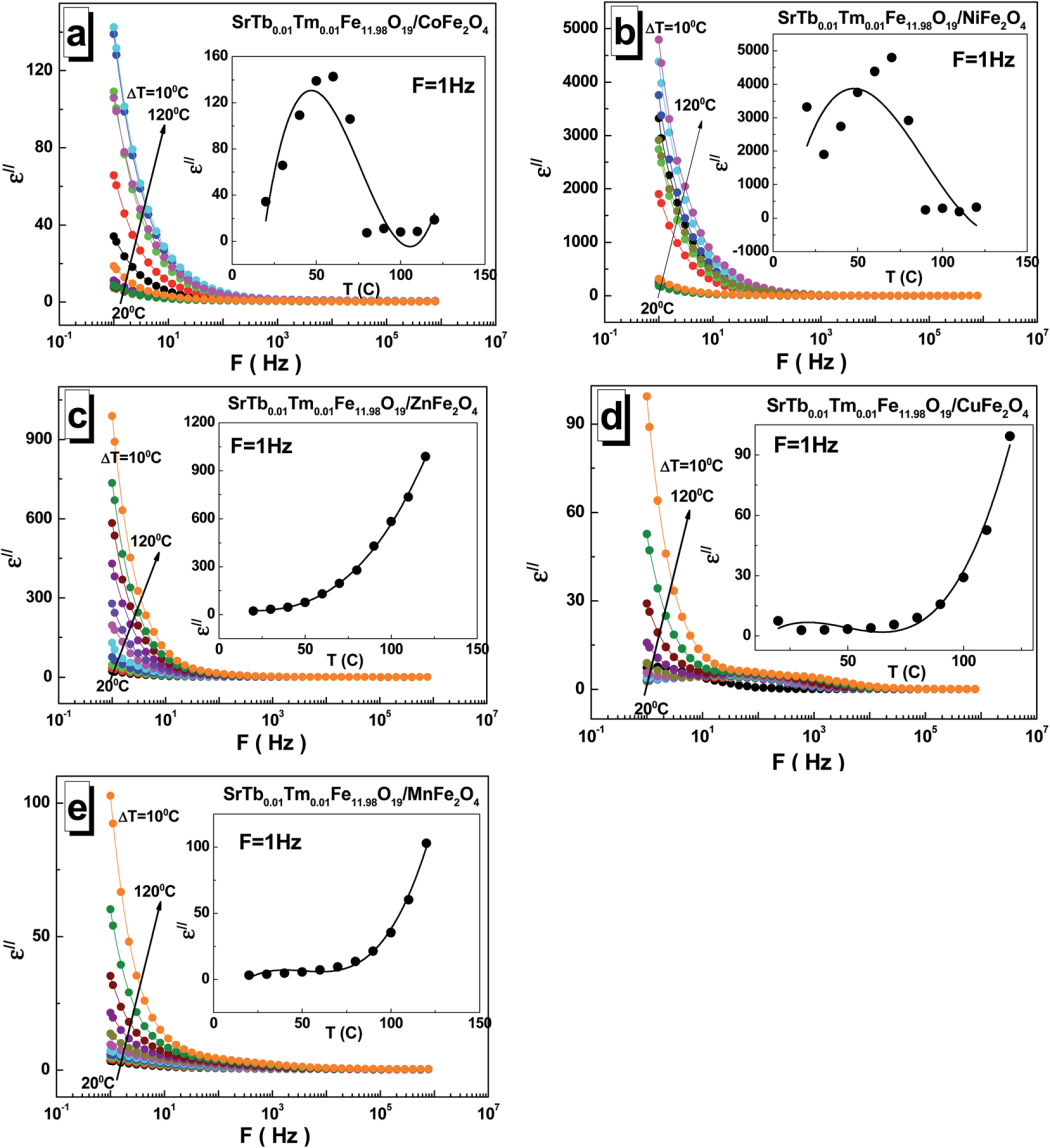
Frequency dependences of the imaginary part *ε*′′ of permittivity in the range of 1–3 × 10^6^ Hz at different temperatures ranging from 20 °C to 120 °C with a step of Δ*T* = 10 °C for the STTFO/AFe_2_O_4_ functional composites, where A = Co (a), Ni (b), Zn (c), Cu (d), and Mn (e). Insets demonstrate the temperature dependence of the *ε*′′ at *F* = 1 Hz.

The dependences of the imaginary part *ε*′′ of permittivity for all the investigated STTFO/AF_2_O_4_ composites are shown in Fig. 5(a–e). The observed value also decreases with frequency and generally increases with temperature. An interesting feature was observed for the samples based on copper cations. The local maxima could be fixed for all temperatures in the frequency range of 10–1000 Hz. A monotonic increase in the imaginary part *ε*′′ of permittivity with temperature was observed for the samples based on the Mn^2+/3+^, Cu^2+^, and Zn^2+^ (see insets of Fig. 5(a–e)). For the sample based on the Ni^2+/3+^ cations, a maximum was observed in the region of 50–80 °C.

An inflection point could be detected in the region of 50–80 °C for the sample based on the Co^2+/3+^ cations. The maximum of the imaginary part *ε*′′ of permittivity was found to be ∼4793 at 1 Hz and 70 °C for the sample based on the Ni^2+/3+^ cations, whereas the minimum value of ∼2.8 was obtained at 1 Hz and 30 °C for the sample based on the Cu^2+^ cations.

**Fig. 6:**
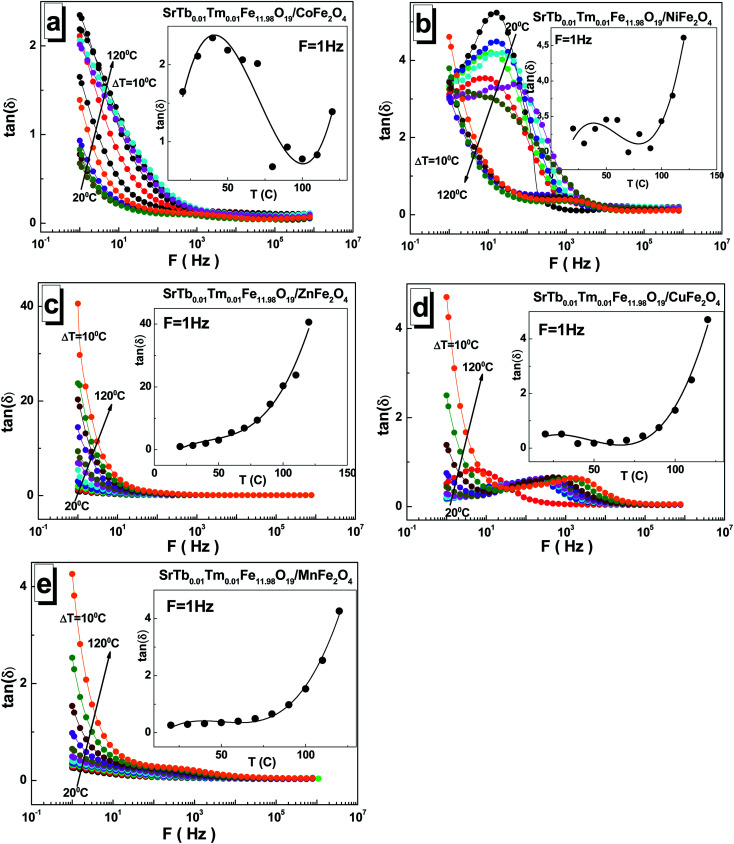
Frequency dependences of the dielectric loss tangent tan(*δ*) in the range of 1–3 × 10^6^ Hz at different temperatures ranging from 20 °C to 120 °C with a step of Δ*T* = 10 °C for the STTFO/AFe_2_O_4_ functional composites, where A = Co (a), Ni (b), Zn (c), Cu (d), and Mn (e). Insets demonstrate the temperature dependence of tan(*δ*) at *F* = 1 Hz.

The values of the dielectric loss tangent tan(*δ*) are relatively low for all the STTFO/AF_2_O_4_ composites. This can be noticed in Fig. 6(a–e). The maximum values reached 4 at low frequencies. An exception was observed for the sample based on the zinc cations. For this sample, the low-frequency value reached 40. The value of the dielectric loss tangent tan(*δ*) also decreased with the increasing frequency. With the increasing temperature, the dielectric loss tangent tan(*δ*) monotonically increased for the samples based on the Mn^2+/3+^, Cu^2+^, and Zn^2+^ cations (see insets of Fig. 6(a–e)). For the samples based on the Co^2+/3+^ and Ni^2+/3+^ cations, an inflection point was observed in the region of 50–80 °C.

The maximum value of ∼41 for the dielectric loss tangent tan(*δ*) was observed at 1 Hz and 120 °C for the sample based on the Zn^2+^ cations. The minimum value of ∼0.17 was obtained at 1 Hz and 40 °C for the sample based on the Cu^2+^ cations.

An interesting feature of the presence of local maxima was noticed for some samples. The occurrence of these maxima in some dependencies can be explained on a qualitative level. Ferrite conduction is controlled by electron hopping between Fe^2+^ and Fe^3+^ cations. At the moment when the frequency of hopping is almost equal to external frequency, maximum energy absorption occurs, causing maximum loss tangent.^39^ The condition for obtaining the maximum tan(*δ*) can be specified by the Debye relaxation relation.^40^ The relaxation time depended on the probability of hopping, and this finding is in good agreement with that reported previously for CoFe_2_O_4_ synthesized using a sol–gel auto combustion method.^41^ Thus, the maximum value of tan(*δ*) can be obtained when the frequency of the electron hopping between the cations Fe^2+^ and Fe^3+^ becomes almost equal to the applied field frequency, and this phenomenon is termed as resonance.

**Fig. 7:**
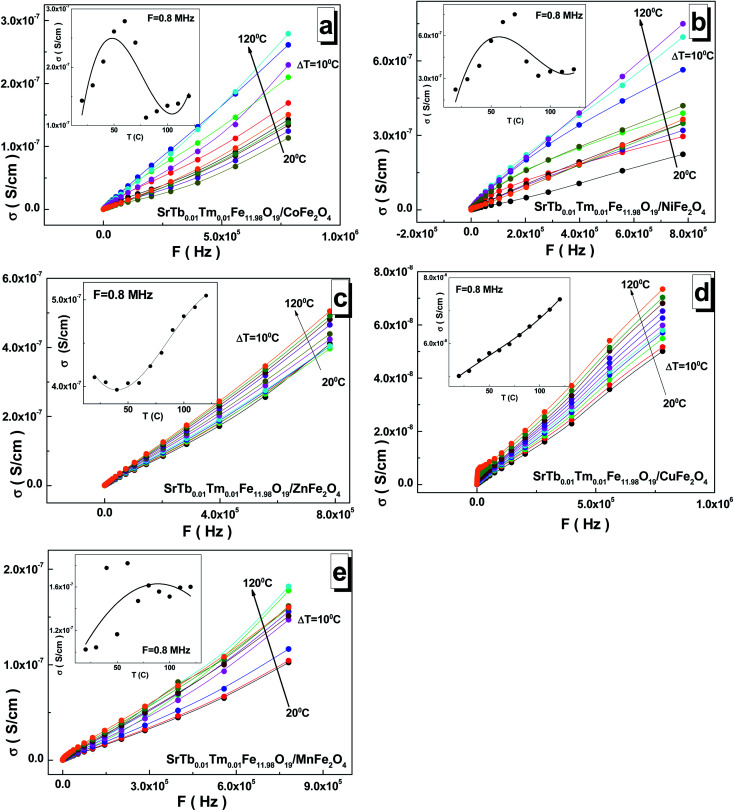
Frequency dependences of the AC-conductivity in the range of 1–3 × 10^6^ Hz at different temperatures ranging from 20 °C to 120 °C with a step of Δ*T* = 10 °C for the STTFO/AFe_2_O_4_ functional composites, where A = Co (a), Ni (b), Zn (c), Cu (d), and Mn (e). Insets demonstrate the temperature dependence of the AC-conductivity at *F* = 0.8 MHz.

The frequency dependences of the AC-conductivity are shown in Fig. 7(a–e). The AC-conductivity steadily increased with the increasing frequency for all the samples. Moreover, it increased with the increasing temperature. In the temperature dependence of the AC-conductivity (see insets of Fig. 7(a–e)), the maximum point was observed for the samples based on the Mn^2+/3+^ and Ni^2+/3+^ cations in the range of 70–100 °C. The minimum point was fixed in the range of 30–50 °C for the sample based on the Zn^2+^ cations. The inflection point was found at 60–80 °C for the sample based on the Co^2+/3+^ cations, whereas a monotonic increase was observed for the sample based on the Cu^2+^ cations. The maximum value of ∼7.50 S cm^−1^ for the AC-conductivity was found at 8 × 10^5^ Hz and 70 °C for the sample based on the Ni^2+/3+^ cations. The minimum value of ∼5.0 S cm^−1^ was obtained at 8 × 10^5^ Hz and 20 °C for the sample based on the Cu^2+^ cations.

As is well-known, AC-conductivity is proportional to external frequency. With an increase in the external frequency, the conductive grains become more momentous; this promotes electron hopping between the two neighbouring iron cations and cause a transition between Fe^2+^ and Fe^3+^, more likely increasing the hopping conduction. Thus, an inevitable increase in the AC-conductivity was observed with the increasing frequency, as reported in the literature.^42^ The AC-conductivity includes two mechanisms. In a high-frequency area, the AC-conductivity mechanism is based on the hopping of electrons, whereas in a low-frequency area, the mechanism is based on the slow motion of electrons. At low frequencies, the AC-conductivity remains almost unchanged. This frequency dependence of the AC-conductivity can be satisfactorily described by Koop's phenomenological theory,^43^ where more conductive grains are surrounded by less conductive boundaries.^44^

### Microwave properties

**Fig. 8:**
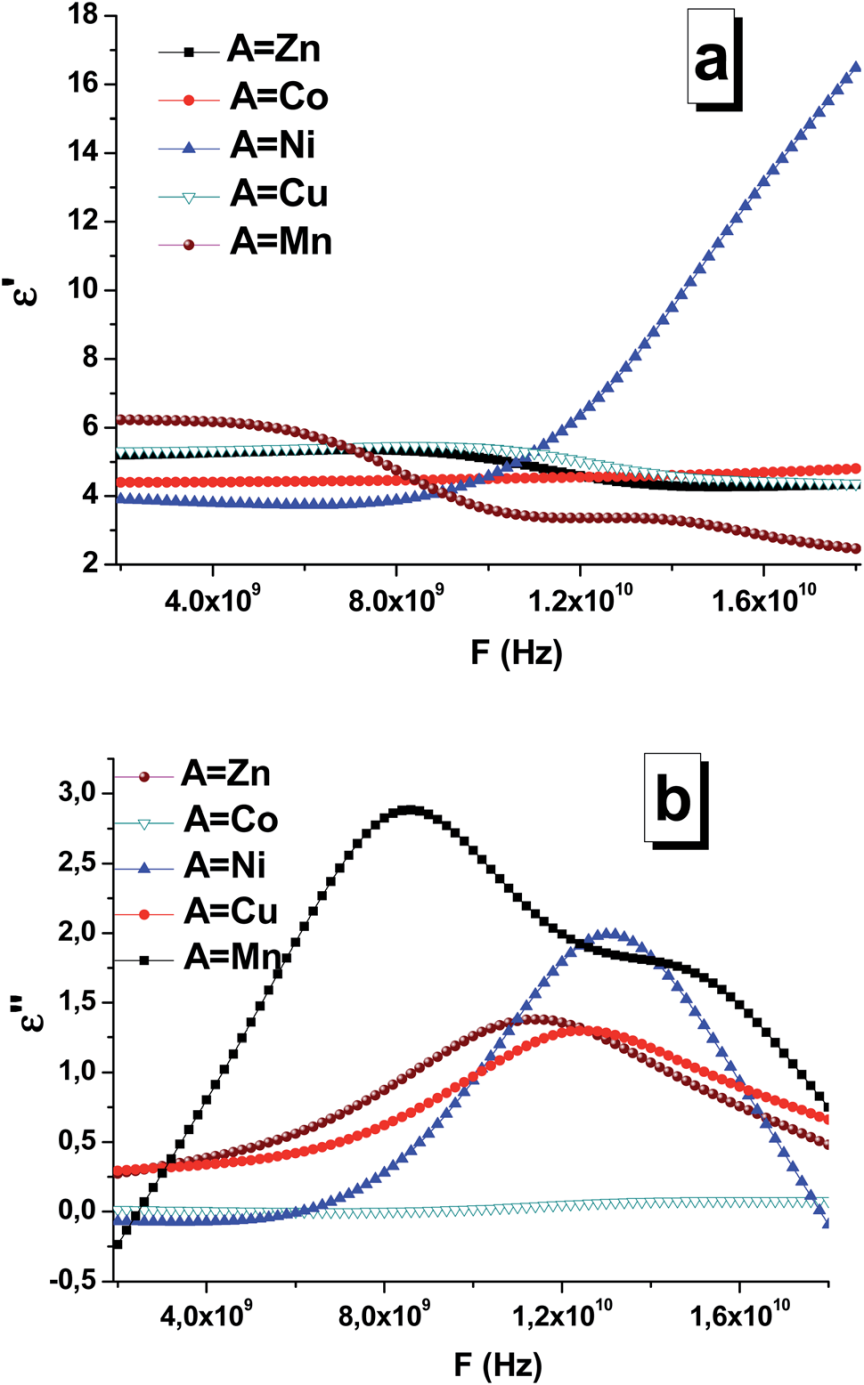
Frequency dependencies of the real (a) and imaginary (b) parts of the permittivity of the STTFO/AFe_2_O_4_ (where A = Co, Ni, Zn, Cu, or Mn) functional composites.

According to the Maxwell's equations, there are two main possible reasons for electromagnetic absorption: electrical losses and magnetic losses. Electrical losses can be observed due to the interaction of the electromagnetic radiation with the charges in material (highly localized and conductive electrons, ions, and dipoles). Magnetic losses can be associated with wave interactions with spin-orbital magnetic moments and domain states. Based on their features and physical nature, electrical losses can be divided into four main types: conductive losses, polarization losses, ionization losses, and losses caused by the heterogeneity of the structure. Thus, three phenomena of the electric field determine or cause energy loss in the dielectric: electrical conductivity, polarization, and ionization. The magnetic losses in complex iron oxides are associated with the excitation of the magnetic state by the external electromagnetic radiation. Magnetic materials, which absorb EMR, convert field energy into heat also due to magnetic losses. If the magnet is in an alternating magnetic field, peaks will possibly be obtained at certain frequencies in the curves of the imaginary part of the magnetic permeability as a function of frequency. In this case, resonance mechanisms of EMR absorption are noticed. Thus, two main types of resonance effects in complex iron oxides can be distinguished: natural ferromagnetic resonance and domain boundary resonance. The dielectric permittivity and magnetic permeability of the STTFO/AFe_2_O_4_ composites (where A = Co, Ni, Zn, Cu, or Mn) were measured using a segment of a coaxial transmission line. Ionic polarization is caused by either the displacement of the nodes of the crystal lattice under the action of an external electric field, where the amount of displacement is less than the value of the lattice constant, or the displacement of the ions weakly fixed at the sites of the crystal lattice. The displacement of ions in the crystal lattice leads to an increase in the polarization vector and an increase in the value of the dielectric constant. The increase in the losses and consequently the increase in the imaginary part of the dielectric constant are associated with the losses caused by the vibration of ions in the crystal lattice. Light elements vibrate with greater amplitude and higher losses, *i.e.* the imaginary part of the dielectric constant, which can be observed in Fig. 8. With an increase in the mass of the elements, the amplitude of the oscillations in the lattice, the value of the losses, and the value of the imaginary part of the dielectric constant decreased. Fig. 8 shows the frequency dependences of the real (Fig. 8a) and imaginary part (Fig. 8b) of the permittivity of the composites. The maximum values (∼3 and 2.2) of the imaginary part of the permittivity were observed for the Mn-based and Ni-based spinels, respectively. Based on the obtained data, it can be established that composition (especially the composition of the soft phase) substantially affects the permittivity. In particular, this was clearly visible for the STTFO/MnFO and STTFO/NiFO samples. For STTFO/CoFO, STTFO/ZnFO, and STTFO/CuFO, the magnitudes of the real part of permittivity are very close. Since there are no peaks for the frequency dependences of the real part of permittivity, the lack of polarization losses in this frequency range for the STTFO/AFe_2_O_4_ composites (where A = Co, Zn, Cu, or Mn) can be considered. The significant increase in the real permittivity of STTFO/NiFO at a frequency above 12 GHz indicates that energy loss occurs during dipole polarization. In Fig. 8b, it can be noticed that the curves exhibit some broad peaks because of the absorption of high-frequency radiation. The position of the peak and the maximum imaginary value of the permittivity are highly dependent on the chemical composition of the STTFO/AFe_2_O_4_ (where A = Co, Ni, Zn, Cu, or Mn) samples. The main limiting parameter is the chemical composition of the soft phase. This may be due to the following reasons: (1) differences in the electron shell configuration and radii of the A-site ions and (2) features of the AC-charge transport due to differences in the microstructure.

**Fig. 9:**
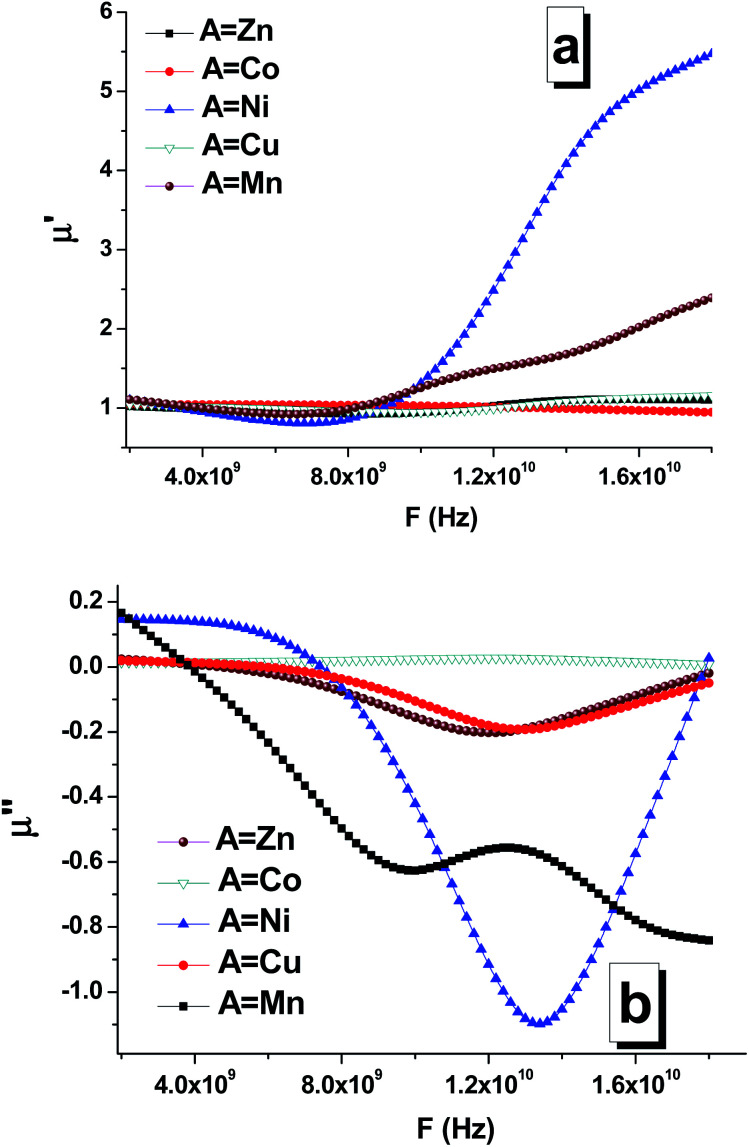
Frequency dependencies of the real (a) and imaginary (b) parts of the permeability of the STTFO/AFe_2_O_4_ (where A = Co, Ni, Zn, Cu, or Mn) functional composites.

The frequency dependences of the real (Fig. 9a) and imaginary parts (Fig. 9b) of the permeability of the composites are shown in Fig. 9. Based on the obtained data, it can be established that the chemical composition (especially the composition of the soft phase) significantly affects the permeability. In particular, this was clearly observed for the real and imaginary parts of the permeability of the STTFO/MnFO and STTFO/NiFO samples. For the STTFO/CoFO, STTFO/ZnFO, and STTFO/CuFO samples, the magnitudes of the real part of permeability are very close. In complex magnetic oxides, there are only a few mechanisms of electromagnetic absorption. Domain boundary resonance (DBR) is one of them. Furthermore, the other mechanism is related to natural ferromagnetic resonance (NFR).

As is known, anomalies in the frequency dependence of the imaginary part of permeability can be determined in the NFR region. Thus, the observed negative values of the imaginary part of permeability can be considered as reflection losses.

Using the calculated values of the real part and imaginary part of the dielectric permittivity and magnetic permeability, the coefficient of reflection for the material layer can be evaluated using the following formula:5
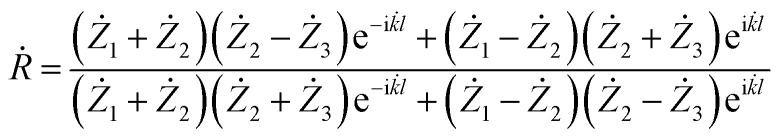
where *Ż*_1_ = wave resistance of the first medium, *Ż*_2_ = wave resistance of the second medium, *Ż*_3_ = wave resistance of the third medium, *l* = the thickness of the material layer of the second medium, and *k̇* = wave number.

We transformed the formula (5) for the case of an electromagnetic wave incident on a layer of material located on an ideally conducting surface.

In this model, we speculate that the electromagnetic wave is incident normally from the medium 3 to the interface between two media. We hypothesize that the wave falls from free space, which means that the resistance of the medium to the resistance of free space is equal to
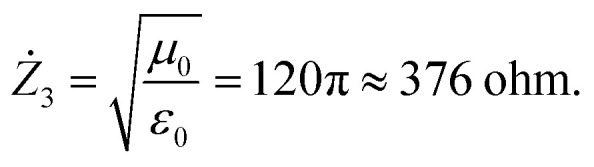


We deduced the resistance of the second medium through the found real part and imaginary part of the dielectric permittivity and magnetic permeability according to the following formula:6
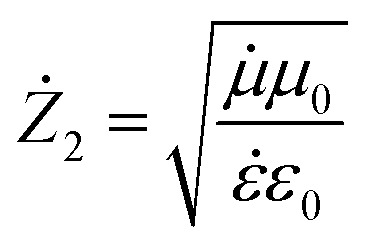


Since medium 1 is perfectly conducting, the resistance of this medium will be equal.

Considering this, we obtained the following formula for the coefficient of reflection from a layer of material on a perfectly conducting surface:7
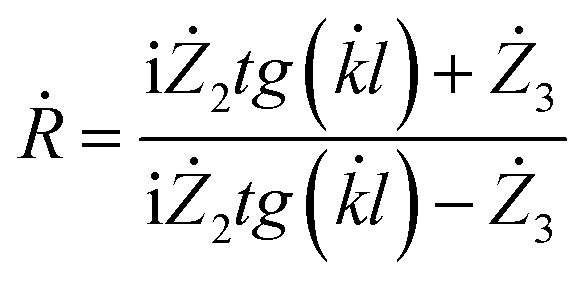


By expressing the resistance through the dielectric and magnetic permeability, we acquired the final formula as follows:8
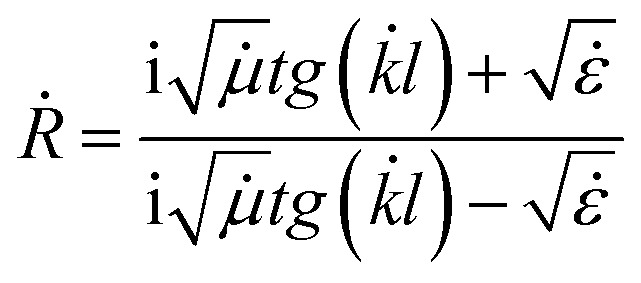


**Fig. 10:**
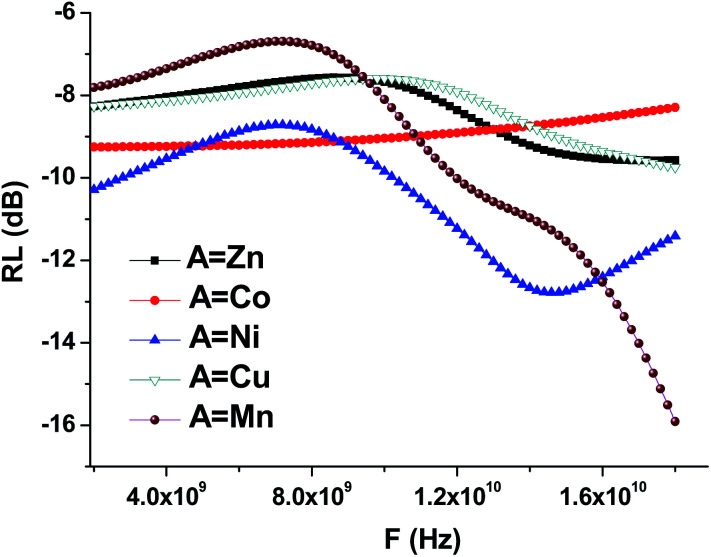
Frequency dependencies of the reflection losses (RL) of the STTFO/AFe_2_O_4_ (where A = Co, Ni, Zn, Cu, or Mn) functional composites.

Using the formula (8), we performed the reflection coefficient calculations for *f* = 2–18 GHz. Fig. 10 shows the frequency dependences of the reflection loss of the STTFO/AFe_2_O_4_ composites (where A = Co, Ni, Zn, Cu, or Mn). Table 4 demonstrates the peculiarities of the main electromagnetic characteristics of the STTFO/AFe_2_O_4_ composites (where A = Co, Ni, Zn, Cu, or Mn).

**Table 4:** Peculiarities of the main electromagnetic characteristics (EMC) of the STTFO/AFe_2_O_4_ (A = Co, Ni, Zn, Cu and Mn) functional composite

EMC	STTFO/AFe_2_O_4_				
	A = Mn	A = Co	A = Ni	A = Cu	A = Zn
*ε*′ (real part)	≈	≈	↑	≈	≈
*ε*′′ (im. part)	↑ max 9 GHz	≈	↑ max 13.5 GHz	↑ max 13 GHz	↑ max 12 GHz
*μ*′ (real part)	↑	≈	↑	≈	≈
*μ*′′ (im. part)	↓ min 9 and 18 GHz	≈	↓ min 13.5 GHz	↓ min 13 GHz	↓ min 12 GHz
Electronic configuration	3d^5^4s^2^	3d^7^4s^2^	3d^8^4s^2^	3d^10^4s^1^	3d^10^4s^2^

At frequencies up to 6 GHz, ionic polarization prevailed in the material samples. Ion polarization occurred due to a shift in the nodes of the crystal lattice as a result of the influence of an external electric field, and the amount of displacement was less than the value of the unit cell parameter. The real part of the dielectric permittivity remained almost constant in this frequency range. Moreover, with the increasing frequency, the imaginary part of the dielectric constant gradually decreased. Fig. 4 shows a similar behavior of the frequency dependencies. The transition from the ionic polarization to the dipole polarization occurs at *F* = 6–8 GHz.

In the frequency range above 8 GHz, dipole polarization prevailed in the material samples. Because of the dipole polarization, which is related to orientation of dipoles in an external field and focused on overcoming the bonding forces inside the atom, high losses are observed at low frequencies. For other materials, the same change in the type of polarization occurred, but in a higher frequency range.^45^

For the Mn, Ni, Cu, Zn materials, losses are caused by an increase in the imaginary part of the dielectric and magnetic permeabilities. The maximum value of the imaginary part of the dielectric constant decreases with an increase in the element (A) serial number for soft magnetic phase (Table 4).

The value of the real part of the magnetic permeability in the 2–10 GHz frequency range is 1, *i.e.* in this range, the material samples behave like diamagnets, as shown in Fig. 9. With the increasing frequency, for materials based on Mn and Ni, the dependence of the real part of magnetic permeability starts to sharply increase. Since the Mn and Ni materials are ferromagnets, their properties are transferred to the samples containing these dopants. This is also confirmed by the fact that for the samples with Mn and Ni, the imaginary part of the magnetic permeability sharply decreases (Fig. 9), and, as a result, the losses in the material decrease also Fig. 10.

For the samples containing Co, Cu, and Zn, the imaginary part remains almost constant in the abovementioned frequency range. This behavior in the frequency range of the magnetic characteristics corresponds to diamagnets, which are Co, Cu, and Zn. Due to this, the loss in the material during the propagation of an electromagnetic wave is substantially less than that for ferromagnets.

## Conclusion

Herein, SrTb_0.01_Tm_0.01_Fe_11.98_O_19_/AFe_2_O_4_ (A = Co, Ni, Zn, Cu, or Mn) functional composites based on SrTb_0.01_Tm_0.01_Fe_11.98_O_19_ hexaferrites as the hexagonal phase and AFe_2_O_4_ ferrite spinel as the cubic magnetic phase with different compositions (different A-site ions) were obtained using a one-pot sol–gel auto-combustion approach. Microstructural analysis demonstrated that the particle size distribution of the STTFO/MnFO sample corresponds to the Gaussian-type function. The other composites, except for the STTFO/MnFO composite, have a bimodal distribution. There are two extreme points of particle size in the graphs. The first extreme point (about 0.1–0.2 μm) corresponds to the contribution of the soft phase. The other extreme point (about 0.8–1.2 μm) corresponds to the contribution of the hard phase or SrTb_0.01_Tm_0.01_Fe_11.98_O_19_. In order to study the dielectric properties of the obtained STTFO/AF_2_O_4_ composite samples, the real *ε*′ and imaginary *ε*′′ parts of permittivity, dielectric loss tangent tan(*δ*), and electrical conductivity at AC in the 1–8 × 10^5^ Hz frequency and 20–120 °C temperature ranges were measured. For the STTFO/AF_2_O_4_ composite samples studied herein, the relaxation of the *ε*′ in the low-frequency range can be considered as a polarization of the grain boundaries. The conduction in ferrites is governed by electron hopping between Fe^2+^ and Fe^3+^ cations. At the moment when the frequency of hopping is almost equal to external frequency, the maximum energy absorption occurs, causing maximum loss tangent. Frequency dependences of the real and imaginary parts of the permittivity and permeability were measured using a segment of the coaxial transmission line in the 2–18 GHz range. Using the measured data, we calculated the reflection losses of the STTFO/AFO samples. At frequencies up to 6 GHz, ionic polarization prevailed in the material samples. Ion polarization occurred due to the displacement of the nodes of the crystal lattice under an external electric field, and the amount of displacement was less than the value of the unit cell parameter. There was a transition from the ionic polarization to the dipole polarization for *F* = 6–8 GHz. At frequencies above 8 GHz, dipole polarization prevailed in material samples. Because of the dipole polarization, which is related to the orientation of the dipoles in an external field and is focused on overcoming the bonding forces inside the atom, high losses are observed at low frequencies. A strong coupling between the hard magnetic (hexagonal) and soft magnetic (spinel) phases was observed. This data provides broad perspectives for practical applications of this kind materials in high-frequency special devices with controllable electromagnetic properties.

## Conflicts of interest

There are no conflicts to declare.

## Supplementary Material
